# Hypoglycemia secondary to type B insulin resistance syndrome

**DOI:** 10.1210/jcemcr/luag008

**Published:** 2026-02-27

**Authors:** Poobalan Naidoo, Fraser James Pirie, Imran Mahomed Paruk

**Affiliations:** Department of Diabetes and Endocrinology, Inkosi Albert Luthuli Central Hospital, Nelson R. Mandela School of Medicine, University of KwaZulu-Natal, Kharwastan 4092, South Africa; Department of Diabetes and Endocrinology, Inkosi Albert Luthuli Central Hospital, Nelson R. Mandela School of Medicine, University of KwaZulu-Natal, Kharwastan 4092, South Africa; Department of Diabetes and Endocrinology, Inkosi Albert Luthuli Central Hospital, Nelson R. Mandela School of Medicine, University of KwaZulu-Natal, Kharwastan 4092, South Africa

**Keywords:** hypoglycemia, type B insulin resistance syndrome, autoimmune, paraneoplastic, immunosuppression

## Abstract

Type B insulin resistance syndrome (TBIRS) is rare, with an unknown prevalence, characterized by antibodies directed against the insulin receptor. TBIRS may present with hypoglycemia and/or hyperglycemia. A case of hypoglycemia secondary to TBIRS is reported. A 24-year-old woman presented with hypoglycemia associated with the adrenergic and neuroglycopenic symptoms. Clinical examination was normal. After workup, including a 72-hour fast, glucagon test, autoimmune screen, computerized tomography of the abdomen and calcium stimulation test, no cause was apparent. The results of the 72-hour fast were equivocal. Given the lack of a diagnosis, antibodies to insulin receptors were measured and found to be positive, confirming TBIRS as the cause. Oral glucocorticoid therapy was initiated, and no further hypoglycemic episodes occurred. Steroid therapy resulted in iatrogenic Cushing syndrome. Steroid therapy was discontinued after 1 year of initiation. She delivered a healthy baby with no neonatal hypoglycemia. Hypoglycemic episodes recurred 3 years later, at which point she had relocated and was treated elsewhere. Although rare, autoimmune causes of hypoglycemia should be considered when other causes are excluded or when results are equivocal. Management with pharmacotherapy can be effective in inducing remission but long-term monitoring is necessary as patients may relapse.

## Introduction

Autoimmune causes of hypoglycemia are rare [1]. There are 2 entities causing autoimmune hypoglycemia, namely insulin autoimmune syndrome (IAS) and type B insulin resistance syndrome (TBIRS) [1]. Autoantibodies may be produced against insulin, as in IAS, or the insulin receptor, as in TBIRS [2].

TBIRS is a rare condition with unknown prevalence [3], characterized by autoantibodies directed against the insulin receptor, which are most commonly of the IgG class [2]. Low titers of insulin receptor antibodies (IRA) are partial agonists of insulin receptors and may cause hypoglycemia through insulin receptor activation [2]. In contrast, high titers of IRA are antagonistic and result in severe insulin resistance and hyperglycemia [2]. Patients with TBIRS, may therefore present with varying symptoms, ranging from hypoglycemia to severe insulin resistance and hyperglycemia [1].

A systematic review which included 119 TBIRS patients with a mean age of 44 years and majority female (76.5%), showed a higher proportion of patients among African Americans (n = 51; 42.9%), followed by Asians (n = 20; 16.8%), Caucasians (n = 11; 9.2%), Latinos (n = 01; 0.8%) and Africans (n = 1; 0.8%) [4]. The most common associated condition was autoimmune disease seen in 52.2%, of which systemic lupus erythematosus (SLE) was most frequent (35.3%). However, in a Japanese cohort of 21 cases, 61.9% of subjects were male and 90.4% were 50 years of age and older [5]. There is no data from Africa.

A definitive diagnosis of TBIRS is based on the presence of detectable IRA. Apart from an association with autoimmune diseases such as SLE, it may also be part of a paraneoplastic phenomenon, for example, secondary to Hodgkin lymphoma and multiple myeloma [2, 5].

An African woman with hypoglycemia secondary to TBIRS is reported.

## Case presentation

A 24-year-old woman with a history of major depressive disorder, presented with a 9-year history of adrenergic and neuroglycopenic symptoms, associated with hypoglycemic episodes. Despite a 9-year history of symptoms, the patient sought hospital care only sporadically and could not provide discharge summaries from her previous admissions and thus details of her hypoglycemic episodes were limited. Hypoglycemic episodes occurred independently of meals, averaging twice per year with moderate severity. Hypoglycemic episodes were self-managed with carbohydrate ingestion. She had no history of using insulin, sulfonylureas, or illicit substances. Her father had type 2 diabetes mellitus and hyperthyroidism. It is uncertain whether the father's hyperthyroidism was autoimmune-related. Clinical examination was normal.

## Diagnostic assessment

During a 72-hour fast, symptomatic hypoglycemia occurred at 15 hours with nadir serum glucose of 50.4 mg/dL (SI: 2.8 mmol/L) (reference range for hypoglycemia, <54 mg/dL [SI: <3 mmol/L]), serum insulin 2.6 mIU/L (SI: 18.1 pmol/L) (reference range for insulin-mediated hypoglycemia ≥3.0 mIU/L [SI: ≥20.8 pmol/L]), C-peptide 0.49 ng/mL (SI: 0.16 nmol/L) (reference range for insulin-mediated hypoglycemia ≥0.6 µg/L [SI: ≥0.2 nmol/L]), cortisol 318 ng/mL (SI: 876 nmol/L) (reference range for insulin-mediated hypoglycemia >199.37 ng/mL [SI: 550 nmol/L]), beta-hydroxybutyrate 26.0 mg/dL (SI: 2.5 mmol/L) (reference range for insulin-mediated hypoglycemia ≤28.1 mg/dL [SI: ≤2.7 mmol/L]) and growth hormone 6.0 ng/mL (SI: 6.0 µg/L) (reference range for insulin-mediated hypoglycemia >3.0 ng/mL [SI: >3.0 µg/L]) (Table 1). A 1-mg glucagon test was performed and serum glucose increased by only 25.2 mg/dL (SI: 0.4 mmol/L) (reference range for insulin-mediated hypoglycemia >30 mg/dL [SI >1.4 mmol/L]) at 30 minutes. Urine sulfonylurea screening was not done due to a laboratory issue. While the insulin-like growth factor (IGF)-1 level was within the normal range at 108 ug/L (SI: 108 nmol/L) (reference range, 107.0-367.0 ug/L [SI 107.0-367.0 nmol/L]), the IGF-2 level was not measured. Kidney and liver function, glycated hemoglobin, lipid profiles, and uric acid level were within normal limits. Adiponectin was not measured. Apart from a positive anti-cyclic citrullinated peptide antibody, there was no laboratory evidence of co-existent autoimmune disease (Table 2). Computerized tomography of the abdomen was normal.

**Table 1:** Results for 72-hour fast

Laboratory test	Reference levels suggesting insulin-mediated hypoglycemia	Patient's result
Plasma glucose	<54 mg/dL [SI: 3 mmol/L]	50.4 mg/dL [SI: 2.8 mmol/L)
Insulin	≥3.0 mIU/L [SI: 20.8 pmol/L]	2.6 mIU/L [SI: 18.1 pmol/L)
C-peptide	≥0.6 µg/L [SI: 0.2 nmol/L]	0.49 µg/L [SI: 0.16 nmol/L]
Cortisol	>199.37 ng/mL [SI: 550 nmol/L]	318 ng/mL [SI: 876 nmol/L]
Growth hormone	>3.0 ng/mL [SI: 3.0 µg/L]	6.0 ng/mL [SI: 6.0 µg/L]
Beta-hydroxybutyrate	≤28.1 mg/dL [SI: 2.7 mmol/L]	26.0 mg/dL [SI: 2.5 mmol/L]

**Table 2:** Autoimmune screen

Test	Result
Anti-cyclic citrullinated peptide antibody	Positive
Anti-nuclear antibody screen	Negative
Double-stranded DNA antibody	Negative
Anti-endomysial antibody	Negative
Thyroperoxidase antibody	Negative
Anti-adrenal antibody	Negative
Islet antigen 2 antibodies	Negative
Anti-parietal cell antibody	Negative
Anti-gliadin IgG and IgA antibody	Negative
Anti-transglutaminase IgG and IgA	Negative

In view of the equivocal biochemical results which partially supported insulin-mediated hypoglycemia, a calcium stimulation test was done, primarily to exclude an occult insulinoma or nesidioblastosis. This showed no increment in hepatic vein insulin levels post-calcium injection into selected abdominal arteries, excluding insulinoma and nesidioblastosis. A diagnosis of TBIRS was made when antibodies to insulin receptors using a radio-receptor immunoprecipitation assay, done in Lübeck, Germany, were found to be positive.

## Treatment

Oral glucocorticoid (prednisone) therapy was initiated at 60 mg daily for 3 months, then tapered over 3 months, and continued on a maintenance dose for a total of 1 year.

## Outcome and follow-up

No further hypoglycemic episodes occurred. However, iatrogenic Cushing syndrome developed. The patient became pregnant 1 year later and delivered a healthy child with no neonatal hypoglycemia. Hypoglycemic episodes recurred 3 years later, at which point she had relocated and was treated in another province.

## Discussion

Although an insulin-mediated cause for hypoglycemia was suspected at presentation, the findings of the 72-hour fast were equivocal. Insulin-mediated hypoglycemia is diagnosed when insulin and C-peptide levels are ≥3.0 miU/L (SI: 20.8 pmol/L) and ≥0.6 µg/L (SI: 0.2 nmol/L), respectively, and the results in this patient were compatible with non-insulin-mediated hypoglycemia. In addition, failure of glucose to rise by more than 25.2 mg/dL (SI: 1.4 mmol/L) following 1-mg glucagon injection was indicative of non-insulin-mediated hypoglycemia. However, a low serum ketone value of ≤15.7 mg/dL (SI: 2.7 mmol/L) was indicative of insulin-mediated hypoglycemia. Furthermore, imaging did not show insulinoma and the calcium stimulation test did not show any evidence to support insulinoma or nesidioblastosis. The presence of IRA confirmed the diagnosis of TBIRS. Sulfonylurea would be an unlikely cause in view of the low insulin and C-peptide results. In this patient, isolated anti-cyclic citrullinated peptide positivity was not linked to clinical evidence of rheumatoid arthritis, although future development of this condition is possible [5].

Patients with TBIRS usually present with hyperglycemia secondary to severe insulin resistance, they require exceptionally high doses of insulin to maintain euglycemia [2, 3]. Furthermore, they tend to have acanthosis nigricans distributed in peri-orbicular, peri-oral, and labial region [2]. Our patient did not experience hyperglycemia or have acanthosis nigricans.

Evidence-based therapeutic options for TBIRS are limited. A retrospective study of 23 patients with TBIRS found that 30% of patients remit spontaneously [3] A National Institutes of Health (NIH) study showed that use of combined immunosuppressive therapy with rituximab, high-dose pulsed steroids, and cyclophosphamide induced remission in 86.4% (n = 19/22) of patients [6]. To maintain remission, azathioprine was utilized [6]. Hypoglycemia was managed through dietary adjustments, specifically frequent meals pairing complex carbohydrates with fats and proteins [7]. Glucocorticoids, such as prednisone, are also used to treat TBIRS, typically starting at a high dose (1 mg/kg, up to 60 mg daily) before tapering to a 5-mg daily maintenance dose [6]. While the specific response rate to corticosteroids remains unknown due to the disease's rarity, and their use carries dose-dependent side effects, this study reported no patient deaths over a 72-month follow-up period [6].

However, the study was limited by being uncontrolled and open label. Furthermore, the costs of these therapies are significant and may limit use in resource limited settings. Cyclophosphamide was not used in the index case because of the risk of ovarian failure, and there was limited access to rituximab.

To the best of our knowledge, and after an extensive literature review, there are no published cases of TBIRS emanating from Africa. However, a case report from France reported on an African woman with TBIRS, HIV, and immune reconstitution syndrome [8]. She was successfully managed with glucocorticoids. The patient described here differed from the French patient of African origin, in that our patient was not exposed to insulin therapy, was not hyperglycemic, and was HIV nonreactive. It is postulated that our patient likely had agonism of the insulin receptor and the French reported patient had antagonism of the insulin receptor, thus accounting for insulin resistance and hyperglycemia.

Reasons for lack of published reports from Africa may be attributed to difficulty accessing IRA assays. In South Africa, there are no laboratories assaying insulin antibodies and was therefore done in done in Lübeck, Germany. In addition, given its rarity, clinicians do not have high suspicion for this condition, and thus tests for antibodies are seldom requested.

## Learning points

- TBIRS is a rare autoimmune disorder that may present with hypoglycemia or hyperglycemia.
- It must be considered in patients with a spectrum of unexplained hypoglycemia, insulin resistance, and hyperglycemia.
- Management with dietary modification and pharmacotherapy can be effective in inducing remission but long-term monitoring is necessary as patients may relapse.
- Pharmacotherapy includes a combination of cyclophosphamide, rituximab, and glucocorticoids.

## Data Availability

Anonymized data supporting the findings of this case report are available from the corresponding author on reasonable request and subject to institutional approval.

## Abbreviations

IRA: insulin receptor antibodies
SLE: systemic lupus erythematosus
TBIRS: type B insulin resistance syndrome
